# Changes in the role of explanatory factors for socioeconomic inequalities in physical performance: a comparative study of three birth cohorts

**DOI:** 10.1186/s12939-021-01592-2

**Published:** 2021-12-11

**Authors:** Silvia S. Klokgieters, Almar A. L. Kok, Marjolein Visser, Marjolein I. Broese van Groenou, Martijn Huisman

**Affiliations:** 1 Department of Epidemiology & Data Science, Amsterdam UMC, Vrije Uniersiteit Amsterdam, Amsterdam Public Health, de Boelelaan 1117, Amsterdam, Netherlands; 2 Department of Psychiatry, Amsterdam UMC, Vrije Universiteit Amsterdam, Amsterdam Public Health, Amsterdam, the Netherlands; 3 Department of Health Sciences, Faculty of Science and Amsterdam Public Health research institute, Vrije Universiteit Amsterdam, Amsterdam, the Netherlands; 4 Department of Sociology, Faculty of Social Sciences, Vrije Universiteit, Amsterdam, The Netherlands

**Keywords:** Socioeconomic inequalities, Physical performance, Cohort differences, Mediation analysis

## Abstract

**Background:**

Due to societal changes and changes in the availability of health promoting factors, explanatory factors of socioeconomic inequalities in health (SIH) may change with time. We investigate differences in the relative importance of behavioural, social and psychological factors for explaining inequalities in physical performance between three birth cohorts.

**Methods:**

Data came from *N* = 988, *N* = 1002, and *N* = 1023 adults aged 55–64 years, collected in 1992, 2002 and 2012 as part of the Longitudinal Aging Study Amsterdam. Physical performance was measured by three performance tests. We included lifestyle factors (physical activity, smoking, alcohol use and Body Mass Index (BMI)); social factors (network size, network complexity, divorce, social support); and psychological factors (mastery, self-efficacy and neuroticism). In multi-group mediation models, we tested whether the strength of indirect effects from socioeconomic position (SEP) via the explanatory factors to health differed between birth cohorts. Stronger indirect effects indicate an increase in the importance; weaker indirect effects indicate a decrease in importance.

**Results:**

Absolute SIH were present and similar across cohorts. The strength of indirect effects of SEP on physical performance through smoking, binge alcohol use, emotional support and mastery increased across cohorts. The indirect effects of BMI, network size, self-efficacy and neuroticism were similar across cohorts.

**Conclusions:**

Inequalities in smoking, binge alcohol use, emotional support and mastery may have become more important for explaining SIH in recent cohorts of middle-aged adults. Policies that aim to reduce socioeconomic inequalities may need to adapt their targets of intervention to changing mechanisms in order to reduce SIH.

**Supplementary Information:**

The online version contains supplementary material available at 10.1186/s12939-021-01592-2.

## Introduction

Recent trend studies have shown that socioeconomic inequalities in physical performance, morbidity and mortality rates have been remarkably persistent in the past decades, despite policy efforts aiming to reduce them [1–4]. The Fundamental Cause Theory [5] posits that factors explaining socioeconomic inequalities in health (SIH) become replaced with others over time due to broad societal developments, resulting in a persistence of SIH [6]. If this is true, policies that aim to reduce SIH would need to be adjusted continually to address the mechanisms that are most important for SIH at a given time.

### Societal changes that may change the explanatory factors of SIH

Throughout the twentieth century, at least three socio-structural changes took place that may affect the relative importance of specific behavioural, social and psychological factors explaining SIH. First, Western countries have entered the third stage of the epidemiological transition; i.e. the stage of ‘diseases of affluence’ [7]. Not only did this development result in an increase in the number of chronic diseases, but also an increase in the number of years individuals are living with lower levels of physical functioning [8]. Because chronic diseases and physical functioning have become more prominent markers of population health, it is likely that differences between socioeconomic groups in health-related behaviours have become more important for explaining SIH as well. For example, studies have shown that factors such as smoking, obesity, and physical inactivity have now become increasingly more prevalent in the lower socioeconomic groups, and that binge alcohol use may have increased predominantly in lower socioeconomic groups [9–12]. Therefore, such behavioural factors may have become more important for explaining SIH in more recent cohorts.

Second, due to processes of detraditionalization and individualization, the influence of traditional sources of social embedding such as the neighbourhood, church and family on social lives has declined [13]. Possible consequences are that ever more people are divorced or remain single [14], and the salience of nonkin in personal networks [13] as well as the overall network size [15] has increased. Individuals with a high socioeconomic position (SEP) may have been better able to draw health benefits and prevent declines in physical performance from these developments, as they historically have had larger and more complex social networks [16]. Therefore, the role of social factors such as network size, network complexity and social support for explaining SIH may also have changed across birth cohorts.

Third, many Western countries have witnessed increased opportunities for upward mobility in the past decades, primarily due to increased access to education. Furthermore, educational and occupational achievement are less dependent on family background but on cognitive skills and effort [17]. Some argue that this shift to a more meritocratic society leads to an increasing selection of individuals with ‘favourable’ personality profiles reflecting a strong sense of control over life and better adaptability to change, as well as with better cognitive skills, into higher socioeconomic groups [7]. As a result, lower socioeconomic groups may have become more homogeneous and disadvantaged regarding such characteristics. If so, psychological factors such as control and cognitive skills may have become more important as explanatory factors of SIH over time.

Building on the observations described above, we investigate whether the roles of social, behavioural and psychological factors for explaining SIH have changed between three cohorts of adults aged 55–64, born in 1928–1937, 1938–1947 and 1948–1957.

We examine cohorts aged 55–64 years, an age-range which represents a moment in the life course where people are often still employed but at the same time start developing health problems, such as chronic diseases and decline of physical function. SIH also tend to be largest around this age, while samples are still minimally biased by selective attrition [18] Finally, it is difficult to find cohort studies that have data from the same independent age groups observed multiple decennia apart. The Longitudinal Aging Study Amsterdam (LASA) is one of the few studies that provides this, yet has no participants younger than 55 years. We use the outcome of physical performance, which is measured by performance tests and is therefore less likely than self-report measures to be influenced by generational changes in the expectations of people for their health and improved detection of diseases [19]. Moreover, it has been shown that older adults who have a lower socioeconomic position (SEP) report poorer physical performance and more disabilities than older adults who have a higher socioeconomic position [20], a pattern that persists across birth-cohorts [4, 21]. Tackling socioeconomic inequalities in physical performance are important because low physical functioning are associated with lower quality of life, a higher need for long-term care, and higher rates of mortality [20].

## Methods

### Design and study sample

LASA is a cross-sequential longitudinal study which focusses on physical, cognitive, social, and emotional functioning among Dutch middle-aged adults [22, 23]. Respondents were randomly drawn from population registers of eleven municipalities in areas covering different levels of urbanicity and religious background. Respondents were visited at home by interviewers who conducted computer-assisted two-hour interviews. The baseline measurement in 1992–93 included respondents aged 55–84, born between 1908 and 1937. In 2002–03 and 2012–13, a new cohorts were added with respondents aged 55–64, born between 1938 and 1947 and between 1948 and 1957, respectively. From the first baseline measurement, we selected only those aged 55–64 years. As such, we were able to compare three cohorts of respondents with the same age, but each observed 10 years apart. We refer to these cohorts as the 28/37-cohort (*n* = 988); the 38/47-cohort (*n* = 1002); and the 48/57-cohort (*n* = 1022). Cooperation rates of the cohorts were 62, 62, and 63%, respectively. LASA was approved by the Ethical Review Board of the VU Medical Center. For more information about the LASA study, we refer to one of the cohort profiles [22, 23] or the webpage (www.lasa-vu.nl/en/).

### Outcome

*Physical performance* was a composite of three performance tests encompassing upper and lower extremity function: a walking test (walk three meters, turn 180° and walk back), chair stand test (stand up and sit down from a chair five times with arms folded) and a cardigan test (take a cardigan on and off). The time needed to complete each test was divided into quartiles, which were assigned a score of 1 (slowest quartile) to 4 (fastest quartile), and 0 for an incomplete test. The final score represented the sum of these scores, ranging from 0 to 12, with higher scores indicating better physical performance. Physical performance is a key indicator of health and functioning in the age-group that we investigate [24].

### Socioeconomic indicators

*Education* was asked in nine levels, which were recoded into nominal years it takes to complete a level. Range was from 5 to 18 (5 = *elementary not completed* to 18 = *university education*).

*Occupation* was based on five skill levels ranging from 1 to 5 (1 = *elementary* to 5 = *scientific*). Of the respondents’ current, previous and longest held occupation, we used the highest reported. Occupations were coded based on the Standard Classification of Occupations 1992 [25].

*Income* included 12 categories ranging from €454–€567 to €2268 or more per month. To make all incomes equivalent to one-person household incomes, the median value of the income category of respondents whose partner contributed to the household income was multiplied by 0.7 [26]. This correction was based on the ratio in Dutch state pensions for citizens living alone and living with others. Missing data was imputed using income data from subsequent measurement waves of LASA. This yielded 151 imputations for the 28/37-cohort, 59 imputations for the 38/47-cohort, and 33 imputations for the 48/57-cohort. Imputed values were adjusted for inflation since the respective cohorts’ baseline measurement [27].

### Lifestyle factors

*Smoking* included two categories: 0 = *never smoker or quit more than 15 years ago* and 1 = *current smoker or quit less than 15 years ago*.

*Binge alcohol use* included two categories:0 = *no alcohol or less than 5 beverages per occasion* and 1 = *5 or more beverages per occasion*.

*Body Mass Index (BMI)* was obtained by dividing measured body weight in kilograms by measured height in meters squared, resulting in a range of 19–45 kg/m^2^.

*Physical activity* used the LASA Physical Activity Questionnaire (LAPAQ) with questions on activities including walking outdoors, bicycling, gardening, light and heavy household activities and a maximum of two sports, in the previous 2 weeks. The minutes spent per activity per day were multiplied by metabolic equivalent (MET) scores appropriate for the intensity of the specific activity [28, 29], and then summed. The physical activity score ranged between 0 and 371 MET-hours/week. Due to non-normality of residuals, the variable was log-transformed.

### Social factors

*Marital status* was based on municipal register data. Two dummy variables were included: ‘divorced’ (1 = *yes*/0 = *no*) and ‘current partner’ (1 = *yes*/0 = *no*) (i.e. widowhood or never having had a partner is the reference group).

*Network size* was based on the question: ‘Name the people you have frequent contact with and who are also important to you’ [30]. Only persons aged 18 and older could be nominated, and all people mentioned were counted (range 0–80).

*Network complexity* included an adapted version of the Social Network Index [31], assessing the number of social roles in which a respondent is involved. Respondents receive a point for each of the following social roles: Spouse, child, child-in-law, sibling, sibling-in-law, parent, (other) relative, close friend, acquaintance, neighbour, (former) colleague, voluntary organization, and other (observed range: 0–11).

*Emotional and instrumental support* were based on the frequency of receiving support from the nine network members indicated as most important by the respondent, except the partner. Questions asked how often during the previous year the respondent had talked to a network member about personal experiences and feelings (emotional support), and how often during the previous year the respondent had received help from a network member with daily chores around the house (instrumental support). The response categories were (0 = *never* to 4 = *often*) and the sum scores for each type of support ranged from 0 to 36.

### Psychological factors

*Crystallized intelligence* (vocabulary) was assessed with a subtest of the Groninger Intelligence Test [32]. Twenty words of increasing difficulty were presented and the respondent had to choose the synonym out of five alternatives. The total score ranged between 0 and 20.

*Mastery* reflects the extent to which a person feels that life circumstances are under one’s own control. We used a five-item version of the Pearlin Mastery Scale [33], including items such as “What happens to me in the future mostly depends on me”. Response categories ranged from 1 (*strongly disagree*) to 5 (*strongly agree*). The total score ranged between 5 and 25, with higher scores representing more internal locus of control.

*Self-efficacy* assessed an individual’s belief in the ability to organize and execute behaviours that are necessary to attain personal goals, and was measured by the 12-item General Self-Efficacy Scale [34]. An example item is ‘when I have decided to do something, I will do it’. Response categories ranged 1 (*never*) to 5 (*very often*), resulting in a total score between 12 and 60.

*Neuroticism* reflects an individual’s proneness to psychological distress [35]. The scale was based on 15 items from the Dutch Personality Questionnaire [36], with response categories 0 (*does not apply to me*) to 2 (*applies to me*). An example item is ‘I am often nervous’. The sum score ranged from 0 to 30.

### Statistical analyses

All analyses were performed in Mplus version 7 [37], using Structural Equation Modelling. Missing variables were handled using full information maximum likelihood (FIML). To measure SEP as a construct consisting of three interrelated indicators, we constructed a latent variable based on education, occupational level and household income [38]. We did so because each indicator may have independent effects on health and the relations between these indicators may have changed between birth cohorts [39]. Using a latent variable accounts for the shared variance between socioeconomic indicators whilst taking into account the unique contribution of each indicator to the underlying construct of SEP. Moreover, it considers differences in the distributions of the indicators between birth cohorts. Information on the statistical fit of the latent variable is presented in the supplementary material Table 1 [40].

We conducted multigroup mediation analysis, modelling psychological, social and behavioural factors as mediators of the relationship between SEP and physical performance. For each explanatory factor and birth cohort separately, we first estimated the total effect of SEP on physical performance (*c* path in Fig. 1, supplementary material) using linear regression. Next, we used linear regression to estimate the direct effects of SEP on the explanatory factor (the *a* path), SEP on physical performance (the *c’* paths), and the explanatory factor on the physical performance (the *b* paths). We calculated indirect effects by taking the product of the effect estimates of the *a* and *b* paths and then estimating 95% confidence intervals around these indirect effects using bootstrapping based on 1000 bootstrap resamples [41]. Age and sex were used as control variables in all models.

Subsequently, we tested whether the strength of direct and indirect effects estimates differed between birth cohorts. Equality of indirect effect estimates between cohorts was tested with a chi square test. If the strength of the indirect effect was stronger in later cohorts, we concluded that the explanatory role of the mediator has become more important, and vice versa for weaker indirect effects. For correctly interpreting such changes, we also examined the two direct effects that jointly constitute the indirect effect (i.e., from SEP to the explanatory factor and from the explanatory factor to health) using a Wald test. This allowed us to establish whether the change of the indirect effect was due to a change in the association between SEP and the mediator, to a change in the association between the mediator an physical performance, or to a change in both.

In order to test whether the explanatory role of the overall set of mediators increased or decreased over time we constructed a final model with multiple mediators. In this model we included all mediators for which we found a statistically significant indirect effect in one or more cohorts (*p* < 0.10). To handle multicollinearity among mediating variables [42], we excluded mediators that were highly correlated with other mediators in favour of the explanatory factor that showed the largest indirect effect (excluded mediators: network complexity and neuroticism). The strength of the total mediation effect was and equality of indirect effects across cohorts were obtained by the same procedures described above.

Due to the technically complex multiple-group mediation setting with bootstrapping procedures, it was necessary to treat the three binary mediators (smoking, binge alcohol use and divorce) as continuous variables in order to estimate the model. Nevertheless, the direction of the effects did not differ between models using logistic and linear regression, we reported the results with linear regression.

## Results

### Descriptive results

Descriptive results are depicted in Table 1. On average, respondents had slightly higher physical performance scores in the two recent cohorts (Table 1). Overall, respondents scored more favourably on all three socioeconomic indicators and had more favourable health behaviours, social network characteristics, psychological characteristics in more recent cohorts. Exceptions were the percentage of individuals who smoked, which decreased across cohorts, the average level of physical activity, which remained stable across cohorts, and the level of emotional support, which was lowest in the 38/47-cohort.

**Table.1:** Characteristics of 54-65 year olds in three birth cohorts (1928-1937, 1938-1947, 1948-1957)

		28/37-cohort	38/47-cohort	48/57-cohort	p Value
	N	M (SD) /%	M(SD) /%	M(SD) /%	t/F test
Age	3013	60 (3)	60 (3)	60 (3)	<0.01
Sex (0-1)					
Women	3013	516 (52)	527 (53)	527 (52)	
Physical performance (3-12)	2815	8 (2)	9 (2)	9 (2)	<0.05
Education (5-18)	3011	9 (3)	10 (3)	12 (3)	<0.001
Occupational level					<0.001
Elementary	224	77 (9)	74 (8)	73 (8)	
Low	800	312 (35)	301 (31)	187 (20)	
Medium	1037	341 (38)	351 (36)	345 (36)	
High	565	113 (13)	195 (20)	257 (27)	
Scientific	185	51 (6)	42 (4)	92 (10)	
Income (317-2439)	2904	1065 (458)	1137 (366)	1615 (408)	<0.001
Behavioural factors					
Smoking (0-1)					<0.05
No or quit >15 years	1489	436 (49)	486 (53)	567 (64)	
yes or quit </= 15 years	1207	454 (51)	433 (47)	320 (36)	
Alcohol (0-1)					<0.001
<5	172	41 (5)	71 (8)	60 (7)	
>=5	2524	849 (95)	848 (92)	827 (93)	
Physical activity score (0-371)^a^	2959	65 (64)	55 (54)	53 (49)	
BMI (19-45)	2658	28 (4)	27 (4)	27 (5)	<0.001
Social factors					
Divorce (0-1)					<0.001
Yes	279	57 (6)	111 (11)	111 (11)	
Partner status (0-1)					
Yes	2361	774 (78)	799 (80)	788 (77)	
Network size (0-80)	2968	15(8)	15 (9)	20 (11)	<0.001
Network complexity (0-11)	2968	5 (2)	5 (2)	6 (2)	<0.001
Instrumental support (0-36)	2961	14 (6)	15 (6)	16 (6)	<0.001
Emotional support (0-36)	2961	23 (8)	22 (8)	23 (7)	<0.01
Psychological factors					
Crystallized intelligence	2855	13 (4)	13 (4)	14 (3)	<0.01
Mastery (8-25)	2971	18 (3)	18 (4)	19 (3)	<0.001
Self-efficacy (23-59)	2977	43 (5)	43 (6)	45 (6)	<0.001
Neuroticism (0-28)	2592	6 (6)	6 (6)	4 (5)	<0.001

^a^due to non-normality median an interquartile range are reported

### Total effect of SEP on health

The total effect of SEP and physical performance was positive, indicating better physical performance with higher SEP (Table 2).

**Table.2:** Total effect model with physical performance and SEP (C)

	Total sample			28/37-cohort			38/47-cohort			48/57-cohort		
	Beta	95% CI		Beta	95% CI		Beta	95% CI		Beta	95% CI	
SEP	0.187	0.153	0.221	0.175	0.112	0.238	0.213	0.150	0.277	0.177	0.120	0.234

Note**.** Adjusted for age and sex

### Behavioural explanatory factors

The indirect effect of SEP via smoking on physical performance was significantly stronger in the 48/57-cohort compared to the 28/37-cohort (28/37-cohort: β = .002, 95% CI [− 0.003, 0.008], 48/57-cohort: β = .019, 95% CI [0.004, 0.034]; Table 3). We found that this increase in the strength of the indirect effect was mainly due to an increase in socioeconomic inequalities in smoking in later cohorts (i.e., the direct effect of SEP on smoking increased; Table 4).

**Table.3:** Indirect effects between SEP, the mediators and physical performance in the total sample and across cohorts

	28/37-cohort			38/47-cohort			48/57-cohort		
Behavioural factors	Beta	95% CI		Beta	95% CI		Beta	95% CI	
Via smoking	0.002^C^	-0.003	0.008	0.004	-0.004	0.012	0.019^A^	0.004	0.034
Via binge alcohol use	-0.001^C^	-0.040	0.028	0.001	-0.027	0.036	0.044^A^	0.005	0.083
Via physical activity	0.001	-0.005	0.006	0.006	-0.004	0.016	0.001	-0.005	0.007
Via BMI	0.017	0.002	0.032	0.014	0.002	0.025	0.028	0.011	0.044
Social factors									
Via divorce	0.002	-0.017	0.022	-0.002	-0.013	0.010	0.007	-0.006	0.020
Via network size	0.014	0.004	0.024	0.008	-0.003	0.019	0.012	0.000	0.024
Via network complexity	0.001	-0.006	0.009	0.001	-0.004	0.006	-0.009	-0.019	0.001
Via instrumental support	0.000	-0.003	0.002	0.004	-0.002	0.010	-0.004	-0.010	0.002
Via emotional support	0.011^C^	0.002	0.021	0.011	-0.003	0.025	0.031^A^	0.016	0.046
Psychological factors									
Via crystallized intelligence	0.044	-0.006	0.095	0.033	-0.025	0.091	0.002	-0.050	0.054
Via mastery	0.014^B,C^	0.008	0.030	0.055^A^	0.034	0.075	0.039^A^	0.020	0.058
Via self-efficacy	0.023	0.008	0.038	0.042	0.022	0.062	0.052	0.030	0.074
Via neuroticism	0.016	0.003	0.028	0.030	0.014	0.046	0.026	0.012	0.040

Note. Adjusted for age and sex, A = different from 28/37-cohort, B = different from 38/47-cohort, C = different from 48/57-cohort

**Table.4:** Direct effects between SEP, the mediators and physical performance (PF) that were different between cohorts

	28/37-cohort			38/47-cohort			48/57-cohort		
	Beta	95% CI		Beta	95% CI		Beta	95% CI	
Behavioural factors									
Smoking									
C’: SEP on PF	0.173	0.110	0.236	0.209	0.146	0.273	0.158	0.098	0.217
B: Smoking on PF	-0.082	-0.142	-0.023	-0.031	-0.087	0.026	-0.083	-0.140	-0.025
A: SEP on Smoking	-0.026^C^	-0.089	0.036	-0.128^C^	-0.193	-0.064	-0.231^A,B^	-0.291	-0.172
Binge alcohol use									
C’: SEP on PF	0.176	0.113	0.239	0.213	0.148	0.277	0.163	0.105	0.221
B: Alcohol on PF	0.009^C^	-0.048	0.066	-0.005^C^	-0.063	0.053	-0.102	-0.159	-0.045
A: SEP on Alcohol	-0.103	-0.166	-0.041	-0.128	0.053	-0.066	-0.132	-0.192	-0.072
Physical activity									
C’: SEP on PF	0.177	0.114	0.239	0.212	0.149	0.274	0.179	0.122	0.236
B: Physical activity on PF	0.080	0.020	0.141	0.135	0.075	0.195	0.099	0.045	0.153
A: SEP on Physical activity	-0.012	-0.071	0.047	0.054	-0.005	0.113	-0.017	-0.074	0.039
BMI									
C’: SEP on PF	0.157	0.092	0.221	0.200	0.136	0.264	0.150	0.091	0.208
B: BMI on PF	-0.087	-0.146	-0.027	-0.090	-0.146	-0.033	-0.121	-0.179	-0.063
A: SEP on BMI	-0.201	-0.265	-0.136	-0.151	-0.216	-0.085	-0.228	-0.288	-0.169
Social factors									
Divorce*									
C’: SEP on PF	0.174	0.111	0.237	0.217	0.154	0.280	0.182	0.125	0.238
B: Divorce on PF	-0.005	-0.064	0.055	0.037	-0.027	0.102	-0.061	-0.119	-0.003
A: SEP on Divorce	0.052	-0.005	0.109	-0.034	-0.088	0.019	-0.055	-0.106	-0.003
Social factors									
Network size									
C’: SEP on PF	0.161	0.098	0.225	0.205	0.141	0.270	0.162	0.106	0.219
B: Network size on PF	0.099	0.045	0.153	0.042^C^	-0.012	0.096	0.195^B^	0.145	0.246
A: SEP on Network size	0.142	0.080	0.204	0.191^C^	0.131	0.252	0.063^B^	0.005	0.120
Network composition									
C’: SEP on PF	0.174	0.111	0.236	0.211	0.148	0.274	0.182	0.126	0.239
B: Network composition on PF	0.116	0.063	0.170	0.069	0.015	0.122	0.157	0.105	0.208
A: SEP on Network composition	0.011	-0.052	0.074	0.018	-0.044	0.081	-0.055	-0.137	0.002
Instrumental support									
C’: SEP on PF	0.175	0.113	0.238	0.211	0.132	0.253	0.181	0.123	0.238
B: Instrumental support on PF	0.019^B^	-0.035	0.073	-0.080^A,C^	-0.137	-0.029	0.057^B^	0.005	0.109
A: SEP on Instrumental support	-0.017	-0.080	0.047	-0.050^C^	-0.100	0.021	-0.068	-0.126	-0.010
Emotional support									
C’: SEP on PF	0.164	0.100	0.227	0.202	0.137	0.267	0.145	0.086	0.204
B: Emotional support on PF	0.084	0.028	0.140	0.051^C^	-0.008	0.110	0.141^B^	0.083	0.198
A: SEP on Emotional support	0.133	0.073	0.193	0.217	0.161	0.273	0.220	0.169	0.272
Psychological factors									
Crystallized intelligence (CI)									
C’: SEP on PF	0.133	0.047	0.219	0.178	0.083	0.272	0.174	0.099	0.249
B: CI on PF	0.073	0.021	0.130	0.050	-0.038	0.138	0.004	-0.074	0.082
A: SEP on CI	0.610^C^	0.565	0.656	0.663^C^	0.621	0.705	0.539^A,B^	0.488	0.589
Mastery									
C’: SEP on PF	0.163	0.100	0.226	0.161	0.096	0.226	0.140	0.081	0.200
B: Mastery on PF	0.147	0.093	0.202	0.213	0.160	0.267	0.147	0.092	0.201
A: SEP on Mastery	0.096^B^	0.033	0.159	0.250^A^	0.189	0.311	0.262	0.207	0.318
Self-efficacy									
C’: SEP on PF	0.154	0.090	0.219	0.172	0.106	0.239	0.125	0.062	0.187
B: Self-efficacy on PF	0.095	0.039	0.151	0.142	0.085	0.199	0.145	0.089	0.201
A: SEP on Self-efficacy	0.239^B,C^	0.179	0.299	0.293^A^	0.235	0.352	0.360^A^	0.308	0.413
Neuroticism									
C’: SEP on PF	0.161	0.097	0.225	0.183	0.118	0.248	0.151	0.094	0.209
B: Neuroticism on PF	-0.096	-0.157	-0.035	-0.149	-0.205	-0.093	-0.154	-0.211	-0.098
A: SEP on Neuroticism	-0.162	-0.233	-0.092	-0.203	-0.266	-0.140	-0.168	-0.230	-0.107

Note. Adjusted for age and sex, A = different from 28/37-cohort, B = different from 38/47-cohort, C = different from 48/57-cohort

Furthermore, the indirect effect via binge alcohol use was significantly stronger in the 48/57-cohort compared to the 28/37-cohort (28/37-cohort: β = −.001, 95% CI [− 0.040, 0.028], 48/57-cohort: β = .044, 95% CI [0.005, 0.083]). Here, we found that the increase in the indirect effect was mainly due to the increasing harmful effect of binge alcohol use on physical performance. We found no cohort differences in indirect effects of SEP via BMI and physical activity.

### Social explanatory factors

The indirect effect of SEP via emotional support on physical performance was stronger in the 48/57-cohort compared to the 28/37-cohort (28/37-cohort: β = −.011, 95% CI [0.002, 0.021], 48/57-cohort: β = .031, 95% CI [0.016, 0.046]). The increase in the indirect effect was mainly due to a stronger direct effect of emotional support on health in the later birth cohort. No cohort differences in the indirect effects of the other social variables were observed.

### Psychological explanatory factors

The indirect effect of SEP via mastery differed significantly between cohorts and was strongest in the 38/47-cohort (28/37-cohort: β = .014, 95% CI [0.008, 0.030], 38/47-cohort: β = .055, 95% CI [0.034, 0.075], 48/57-cohort: β = .039, 95% CI [0.020, 0.058). The increase in the indirect effect was due to an increase in socioeconomic inequalities in mastery (direct effect SEP on mastery); there was no consistent increase in the effect of mastery on physical performance. Although significant indirect effects via self-efficacy and neuroticism were found in all cohorts, cohort differences in the strength of these indirect effects were not statistically significant. For crystalized intelligence, no significant indirect effects were found in the cohorts.

### Multiple-mediator model

In our final model, we included smoking, binge alcohol use, BMI, network size, emotional support, mastery and self-efficacy (supplementary material Table 2 and Fig. 2). Comparing the cohorts, we found that the sum of indirect effects of this set of mediators increased from the 28/37-cohort to the 38/47-cohort and to the 48/57-cohort (28/37-cohort: β = .059, 95% CI [0.032, 0.087], 38/47-cohort: β = .101, 95% CI [0.060, 0.141], 48/57-cohort: β = .150, 95% CI [0.105, 0.195), thus suggesting more explanatory power in the more recent cohorts.

## Discussion

This study examined changes in the contributions of behavioural, social and psychological mechanisms to SIH across three cohorts of Dutch individuals aged 55–64, born in 1928–1937, 1938–1947 and 1948–1957, respectively. In line with other studies [1–3], we found that SIH were of similar magnitude in all three cohorts. However, we found evidence that smoking, binge alcohol use, emotional support and mastery became more important for explaining these SIH in later cohorts. The importance of several other factors, including BMI, network size, and self-efficacy, remained stable across cohorts. Furthermore, the total contribution of all factors to explaining SIH was larger in more recent cohorts.

Relative to individuals with a low SEP, individuals with a high SEP were less likely to smoke in more recent cohorts and binge drinkers were less likely to report a favourable physical performance, which increased the importance of these mechanisms for explaining SIH. We observed that emotional support increasingly mediated between SEP and physical performance across birth cohorts. While there are studies confirming that middle-aged adults gain increasing levels of emotional support across birth cohorts [13], none have shown that this has an influence on SIH before. We found that instrumental support was inversely associated with physical performance in the 38/47-cohort. It might be that instead of prohibiting poor physical performance, instrumental support reflects a need for assistance with daily tasks [43]. Finally, we found that the mediating role of mastery increased across cohorts, which has not been observed in previous studies before, as far as we know.

We found no evidence for the fact that factors became less important across birth cohorts. Instead, we found that BMI, network size, and self-efficacy remained stable across cohorts. Apparently, individuals with a high SEP have maintained their relative advantage in BMI, network size and self-efficacy over individuals with a low SEP across birth cohorts. This continued importance may be because both individuals with a low SEP and high SEP have equally benefited from increased knowledge about preventive medicine, redistributive social security systems, universal education and health coverage.

Taken together, the full mediation model indicated that the explanatory role of the mediators (i.e. smoking, alcohol, network size, emotional support, mastery and self-efficacy) increased across cohorts. Even after this adjustment, the mediators, the SEP variable retained its significant effect. This means that even though these mediators were able to explain a large part of the relationship between SEP and physical functioning, they were not able the explain the effect in full. Prior studies have similarly shown the importance of behavioural, social and psychological mechanisms in explaining SIH [6]. Of course, these behavioural and psychosocial factors are likely to mutually influence one another. For example a higher sense of mastery may lead more effective behaviour when dealing with problems in social relationships, and reduce health damaging behaviours such as smoking that may increase in stressful situations. As such, it is difficult to infer the contribution of individual mediators based on this model.

### Strengths and limitations

Unlike many other studies that have investigated the contribution of explanatory pathways to SIH by using series of separate regression models [44], we used structural equation modelling to explicitly model the assumed indirect relationships between SEP, explanatory factors and health [42]. Using this approach, we were able to compare the strength of explanatory mechanisms between cohorts, and investigate whether these changes were mainly due to changes in the distribution of explanatory factors between socioeconomic groups, to changes in the effect of explanatory factors on health, or to both. We were able to use a dataset with access to a broad range of explanatory factors and three independent groups of adults of the same age but observed in different years.

Three limitations need to be mentioned. First, we used three cohorts that were born across 29 years (i.e. 1928–1957). Because of this relatively short timeframe, it is possible that some of the long-term consequences of sociostructural changes have not been fully observed. However, it should be noted that cohort-sequential studies such as ours, with ample information on a broad range of potential explanatory factors of SIH, spanning behavioural, social and psychological factors, are rare. Second, it is possible that period, cohort and age effects all contribute to the effects that we found [45], Nevertheless, it is likely that the societal developments that provide the background to our analysis likewise represent a mixture of cohort and period effects. Even if it would be analytically possible, it may not be necessary to differentiate between period and cohort effects in the explanatory mechanisms because we are interested in the possibility that mechanisms of SIH become replaced over time regardless of whether they are a consequence of period or cohort effects. Third, our estimation of the direct and indirect effects with smoking, binge alcohol use and divorce was based on linear rather than logistic or probit regression. Ideally, we estimated models using a combination of linear and logistic regression for the dichotomous mediators. However, this posed statistical problems in terms of comparability of the different paths in the model, and interpretation of indirect effects. Although we explored several possibilities offered by the counterfactual mediation framework to tackle this issue (including transforming odds ratio’s to risk ratio’s) [46], our research question and best practice for mediation modelling required applying these transformations in a multiple group model with a latent SEP variable, parallel mediation, and bootstrapping indirect effects, which was not technically possible. To our knowledge, no alternative solutions with this particular configuration has been described in the literature. This is an important area for methodological refinement of multiple group mediation analysis for future studies.

## Conclusion

We conclude that the explanatory mechanisms of socioeconomic inequalities in physical functioning indeed change over time. More specifically, we found the explanatory roles of smoking, binge alcohol use, emotional support and mastery in socioeconomic inequalities in physical functioning were more important in recent birth cohorts than in earlier birth cohorts. The roles of BMI, network size, social efficacy and neuroticism remained important for explaining SIH. As such, we conclude that the explanatory mechanisms of SIH span across multiple domains (behavioural, social and psychological) are not fixed, but at least partly changed across the past decades. Our results suggests that it is important for researchers to continue to investigate mechanisms of SIH across time to keep the knowledge up to date and to gain insight into how (features of) contemporary societies may be reshaping socioeconomic inequalities. After all, if explanatory mechanisms are prone to change over time, this implies that policies aiming to reduce SIH need to be properly contextualized. Based on our findings, we recommend that researchers and policymakers take into account the socio-structural context and socio-emotional factors in tackling SIH, in addition to a lifestyle oriented approach.

## Supplementary Information

**Additional file 1.**

**Additional file 2.**

**Additional file 3.**

**Additional file 4.**

## Data Availability

Access to data from the Longitudinal Aging Study Amsterdam can be requested by submitting a LASA analysis proposal form for evaluation. The LASA evaluation committee provides access to the data on the condition that the goals of the data request are in keeping with the overarching aims of LASA that its participants have provided consent for. The LASA analysis proposal template includes the option to request data for replication purposes. The template of the analysis proposal form can be obtained at www.lasa-vu.nl, or by sending a request to the LASA secretariat, f.kursun@amsterdamumc.nl. Analysis proposals can be submitted to the LASA secretariat.
