# Supplementary material for: Changes in the role of explanatory factors for socioeconomic inequalities in physical performance: a comparative study of three birth cohorts
Source: Int J Equity Health. 2021 Dec 11;20:252. doi: 10.1186/s12939-021-01592-2 (PMC8665629; doi:10.1186/s12939-021-01592-2)
Supplement: Supplementary file 1 — Additional file 1. [file 12939_2021_1592_MOESM1_ESM.docx]

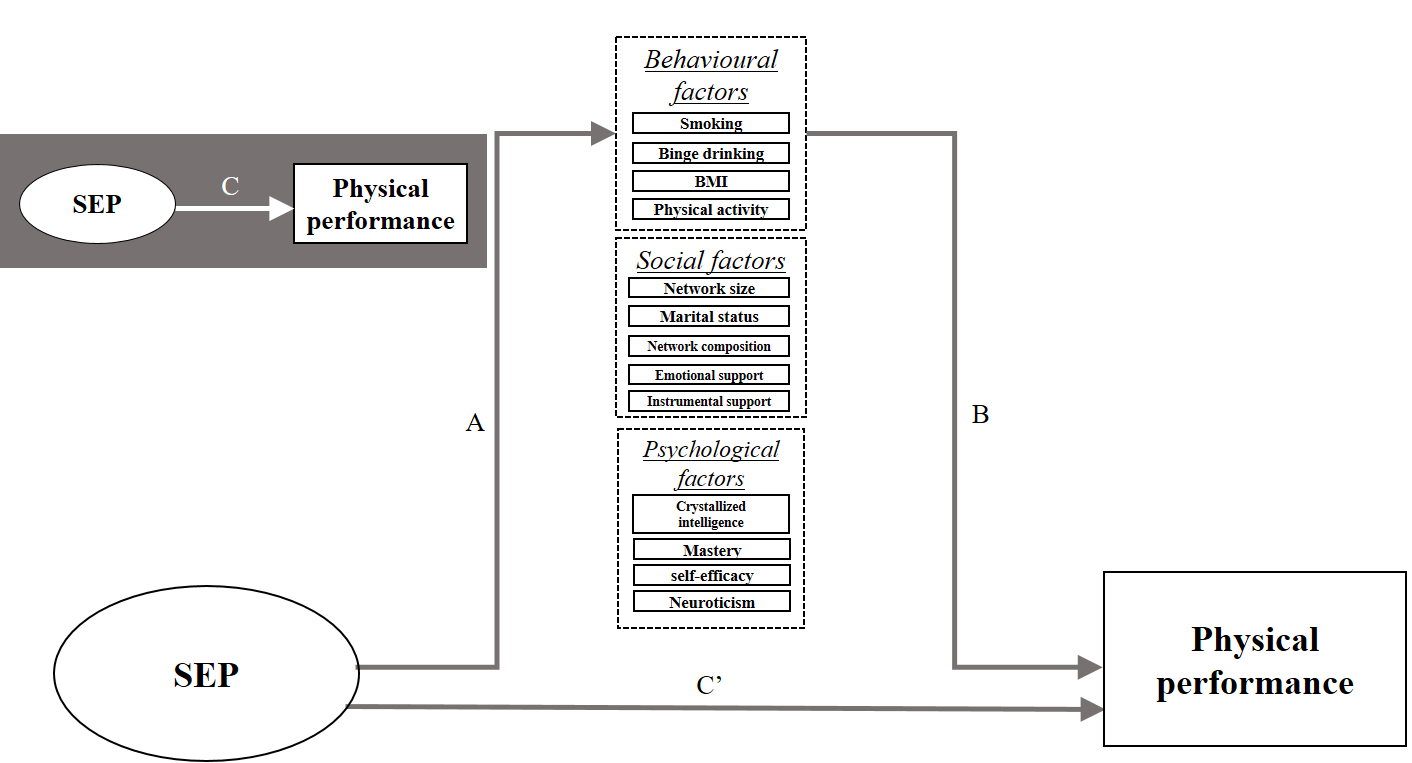


**Figure 1.** Visual presentation of second step in the analysis: testing for all mediators individually mediator
