# Supplementary figures and images for: Changes in the role of explanatory factors for socioeconomic inequalities in physical performance: a comparative study of three birth cohorts
Source: Int J Equity Health. 2021 Dec 11;20:252. doi: 10.1186/s12939-021-01592-2 (PMC8665629; doi:10.1186/s12939-021-01592-2)

**
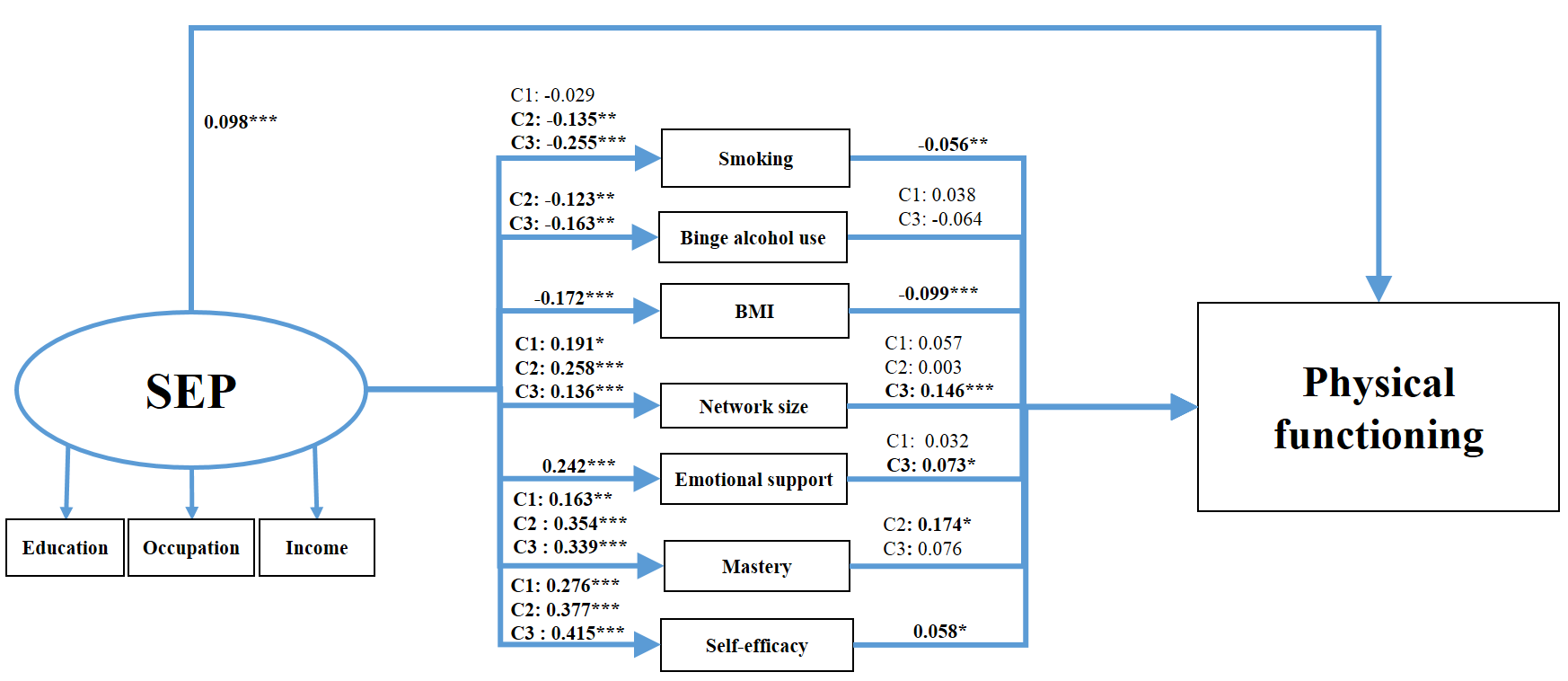
**

**Supplementary Figure 2.** Causal pathways between SEP, mediators and physical performance

Supplement: Supplementary file 2 — Additional file 2. [file 12939_2021_1592_MOESM2_ESM.docx]
