# Supplementary material for: Changes in the role of explanatory factors for socioeconomic inequalities in physical performance: a comparative study of three birth cohorts
Source: Int J Equity Health. 2021 Dec 11;20:252. doi: 10.1186/s12939-021-01592-2 (PMC8665629; doi:10.1186/s12939-021-01592-2)
Supplement: Supplementary file 3 — Additional file 3. [file 12939_2021_1592_MOESM3_ESM.docx]

*Latent SEP variable*

Factor loadings for the latent construct SEP were as follows (Table 2)—education: 0.835 (*p* < .001) (28/37-cohort), 0.761 (*p* < .001) (38/47-cohort), 0.850 (*p* < .001) (48/57-cohort), occupational skill level: 0.594 (*p* < .001) (28/37-cohort), 0.650 (*p* < .001) (38/47-cohort), 0.517 (*p* < .001) (48/57-cohort), and income: 0.729 (*p* < .001) (28/37-cohort), 0.713 (*p* < .001) (38/47-cohort), 0.766 (*p* < .001) (48/57-cohort). No differences in factor loadings were observed between cohorts and the internal consistency of the latent SEP construct was satisfactory in all cohorts (0.77, 0.75, 0.76). This means that the extent to which the individual indicators indicate an latent SEP construct is acceptable within all cohorts.

**Supplementary Table 1.** Factor loadings of latent SEP variable, within and between cohorts

|  | 28/37-cohort | | 38/47-cohort | | 48/57-cohort | |
| --- | --- | --- | --- | --- | --- | --- |
|  | estimate | P | estimate | P | estimate | P |
| Education | 0.835 | *** | 0.761 | *** | 0.850 | *** |
| Occupation | 0.594 | *** | 0.650 | *** | 0.517 | *** |
| Income | 0.729 | *** | 0.713 | *** | 0.766 | *** |
| Composite reliability | 0.77 |  | 0.75 |  | 0.76 |  |
