# Supplementary material for: Changes in the role of explanatory factors for socioeconomic inequalities in physical performance: a comparative study of three birth cohorts
Source: Int J Equity Health. 2021 Dec 11;20:252. doi: 10.1186/s12939-021-01592-2 (PMC8665629; doi:10.1186/s12939-021-01592-2)
Supplement: Supplementary file 4 — Additional file 4. [file 12939_2021_1592_MOESM4_ESM.docx]

**Supplementary Table 2.** Indirect effects between SEP, the mediators and physical performance in the in the path model

|  | 28/37-cohort | | | 38/47-cohort | | | 48/57-cohort | | |
| --- | --- | --- | --- | --- | --- | --- | --- | --- | --- |
|  | B | 95% CI |  | B | 95% CI | | B | 95% CI | |
| Behavioural factors |  |  |  |  |  |  |  |  |  |
| Via Smoking | 0.003 | -0.004 | 0.009 | 0.004 | -0.004 | 0.012 | 0.010 | -0.006 | 0.026 |
| Via Alcohol | -0.005 | -0.012 | 0.003 | 0.001^C^ | -0.007 | 0.010 | 0.010^B^ | -0.002 | 0.023 |
| Via BMI | **0.018** | 0.004 | 0.031 | **0.013** | 0.001 | 0.024 | **0.029** | 0.012 | 0.047 |
| Social factors |  |  |  |  |  |  |  |  |  |
| Via Network size | **0.011** | -0.001 | 0.023 | 0.001 | -0.014 | 0.015 | **0.020** | 0.007 | 0.033 |
| Via Emotional support | 0.006 | -0.006 | 0.017 | 0.007 | -0.010 | 0.023 | **0.018** | 0.002 | 0.034 |
| Psychological factors |  |  |  |  |  |  |  |  |  |
| Via Mastery | **0.017** | 0.004 | 0.031 | **0.062** | 0.034 | 0.089 | **0.026** | 0.002 | 0.049 |
| Via Self-efficacy | 0.010 | -0.009 | 0.029 | 0.013 | -0.014 | 0.040 | 0.037 | 0.008 | 0.066 |
| Sum of indirect effect | **0.059**^C^ | 0.032 | 0.087 | **0.101^C^** | 0.060 | 0.141 | **0.150^A,B^** | 0.105 | 0.195 |

Note. A = different from 28/37-cohort, B =different from 38/47-cohort, C = different from 48/57-cohort
